# Glycemic Control for Type 2 Diabetes Mellitus Patients: A Systematic Review

**DOI:** 10.7759/cureus.26180

**Published:** 2022-06-21

**Authors:** Saud A Bin Rakhis, Nawaf Mohammed AlDuwayhis, Naif Aleid, Abdullah Nasser AlBarrak, Abdullah Ahmed Aloraini

**Affiliations:** 1 Family Medicine, Prince Mohammed Bin Abdulaziz Hospital, Riyadh, SAU; 2 Family Medicine, King Saud Medical City, Family Medicine Academy, Riyadh, SAU

**Keywords:** t2dm, glycemic control, factors, determinants, diabetes mellitus

## Abstract

Diabetes mellitus is a chronic metabolic disorder resulting in hyperglycemia and microvascular and macrovascular complications in individuals globally. Type 2 diabetes mellitus (T2DM) is highly prevalent and accounts for 90% of patients. Maintaining blood glucose concentration is essential to avoid severe complications.

Glycemic control is the optimal serum glucose concentration in diabetic patients. It is necessary to identify factors affecting the glycemic control of patients to prevent control and complications. We conducted this systematic review to assess the factors affecting glycemic control among type 2 diabetes mellitus patients.

Published literature between the years 2020 to 2022 was retrieved from PubMed, Science Direct, and Google Scholar using different combinations of keywords: T2DM, Glycemic control, Poor, Good, Adequate, Inadequate, Factors, Association, and Determinants. All original articles written in the English language with full-text available and the value of glycemic control defined were included. A total of 1866 studies were retrieved. After the title, abstract, screening, and full-text screening, 12 studies were eligible.

The prevalence of poor glycemic control was high, and it ranged between 45.2% and 93% among the studies. The factors associated with glycemic control were stratified into four categories: personal or body-related, clinical, medication-related, and behavioral factors. There was a high prevalence of poor glycemic control in all included studies. The glycemic control was associated with various factors; some were related to the patient or medical conditions while others were related to the behavior of the patients or the medication administrated.

## Introduction and background

Diabetes mellitus (DM) is a rapidly growing public health crisis globally with a huge burden of disease [1]. It is a prevalent metabolic disorder characterized by a deficiency in the secretion of insulin or in its effect or both [2]. In 2019, it was estimated that 463 million individuals are suffering from diabetes, and it is expected to rise to 578 million patients by 2030 and 700 million by 2045 [3]. Type 2 diabetes mellitus (T2DM) is characterized by the failure of beta-pancreatic cells and peripheral insulin resistance [4]. T2DM represents 90%-95% of the overall diabetic cases and despite its global attention and efforts by the healthcare community, its incidence and prevalence continue to rise [5]. New methods of assessing glycemic control are under evaluation nowadays.

For the management of all diabetic patients, the key therapeutic goal is to maintain good glycemic control (GC) in order to prevent macro and microvascular complications [1]. GC is the optimal blood sugar level in a DM patient [6]. Glycemic control in T2DM patients can be evaluated using three parameters: glycosylated hemoglobin (HbA1c), fasting blood glucose (FBG), and postprandial glucose (P PG). Among these, glycosylated hemoglobin is the gold standard for the estimation of glycemic control [7]. The American Diabetes Association (ADA) defines good diabetic control at a cutoff of glycated hemoglobin (Hb1Ac) 7%, whereas the American College of Endocrinologists set it at 6.5%. Regarding fasting blood glucose, the recommended range is 70-130mg/dL (3.9-7.2mmol/l) as set by ADA, whereas the American College of Endocrinologists and the International Diabetes Federation set it at less than 110 mg/dL (6.1 mmol/l) and 100 mg/dl (5.5 mmol/l), respectively [8].

Inadequate glycemic control led to uncontrolled diabetes, which leads to many complications of diabetes mellitus. These complications, in turn, can greatly reduce the quality of life of patients, reduce the life expectancy, as well as increase the healthcare costs of the disease [9-10]. Rigorous recording and controlling of the level of blood glucose is essential to diabetes care and management in order to delay and reduce the incidence of complications [11]. On the other hand, improving glycemic control reduces morbidity and increases the life expectancy and quality of life of patients [12].

Despite its importance, GC compliance has been found to be low due to multiple factors [13]. The identification of factors influencing the GC is crucial to institute appropriate intervention for the improvement of GC [14]. This systematic review identified the factors affecting glycemic control among T2DM patients.

## Review

Method and search strategy

This systematic review was reported in accordance with the Preferred Reporting Items for Systematic Reviews and Meta-Analysis (PRISMA) guidelines [15]. Published literature was retrieved from PubMed, Science Direct, and Google Scholar using different combinations of keywords: T2DM, Glycemic control, Poor, Good, Adequate, Inadequate, Factors, Association, and Determinants.

Eligibility criteria

**Figure 1:**
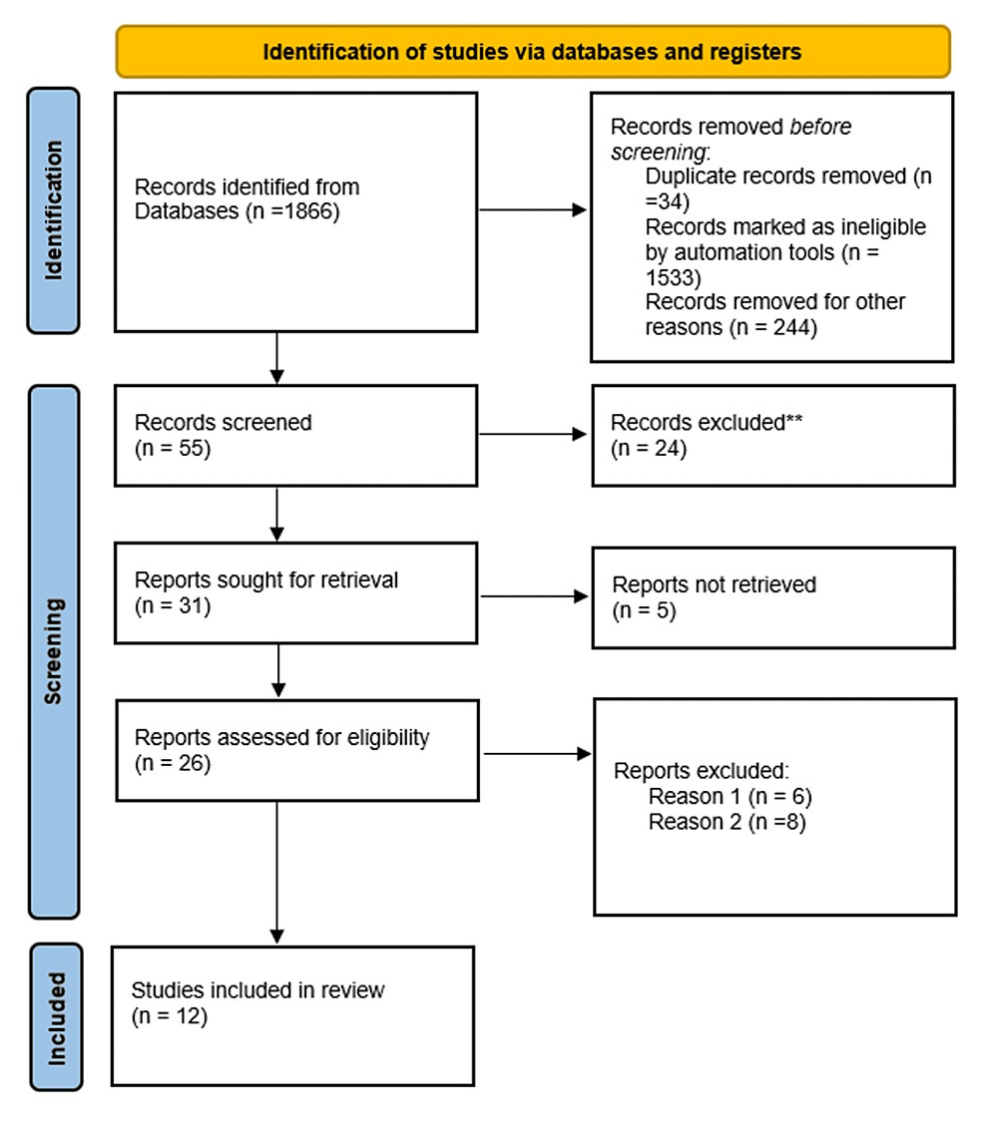
PRISMA flow diagram PRISMA: Preferred Reporting Items for Systematic Reviews and Meta-Analysis **non-original articles Reason 1: Non-full-text articles Reason 2: Articles that didn’t determine glycemic control

Studies were retrieved and two independent authors excluded studies after a title and abstract screening. Articles with titles not focusing on our subject were excluded. This exclusion involved titles studying patients with type 1 diabetes mellitus or both patients with type 1 and type 2 diabetes. All studies published before 2020 were also excluded and only articles that were published between 2020 and 2022 were included. Articles written in English and original articles were included, whereas articles written in a non-English language, review articles, systematic reviews, and letters to the editor were all excluded. Original articles written in English were further reviewed; duplicate articles and non-full text articles were excluded. Also, articles that didn't define or determine glycemic control in their study were excluded. Therefore, the final analysis included original English articles with full-text and duplicate data, which determined the value at which glycemic control was considered good or poor. The full description of the search strategy is shown in Figure 1.

Data review and analysis

Data extraction was performed using a structured Microsoft Excel 2016 worksheet (Microsoft Corporation, Redmond, WA). Variables extracted included author and publication year, study design and study country, sample size, age of participants, gender distribution, the value of glycemic control at which it was considered adequate or inadequate, results and main findings related to the prevalence of poor and good glycemic control, as well as the factors affecting glycemic control. The extracted data were revised through the Excel sheet. The extracted data was transferred to a table.

Results

This systematic review included 12 articles that met the eligible criteria (Table 1) [16-27]. The included articles were published either in 2021 [16-22] or in 2020 [23-27]. No articles were published in 2022. The design of studies was commonly cross-sectional. There were 10 cross-sectional studies [16,18-25,27], whereas the remaining two studies were either retrospective observational [17] or retrospective [26]. Among the cross-sectional studies, there were seven studies that reported a cross-sectional design only [18-20,22-23,25,27], whereas the remaining three studies reported that the studies were prospective cross-sectional [16], descriptive-analytical cross-sectional [21], and descriptive cross-sectional [24].

**Table 1:** Summary of included studies GC; glycemic control, N; number, ADA; American Diabetic Association, FBG; fasting blood glucose

Author and Publication year	Study design	Country	Sample size	Age	Male: N (%)	Glycemic control (GC)	Medications	Results and main findings
Bereda & Bereda 2021 [14]	Prospective cross-sectional	Ethiopia	122	Age: ˃40 years= 66 (54.1%)	67 (54.9%)	GA based on ADA; good GC with FBG of 70-130mg/dl, poor GC with FBG of ˂70 and ˃130mg/dl	Metformin, Glibenclamide and metformin, Glibenclamide, Metformin and NPH insulin, Metformin + Glibenclamide + insulin	The overall incidence of poor glycemic control among type 2 diabetic patients was 60.7%.
								Poor glycemic control was significantly associated with older age (P=0.034), uneducated patients (p=0.009), Glibenclamide + metformin drug regimen (p=0.018), low adherence (p=0.002), cigarette smokers’ social habit (p=0.008), patents who had comorbidities (p =0.028), and nephropathic complication of diabetes(p=0.005) were the significantly associated predictors of poor glycemic control.
Rashad et al 2021 [15]	Retrospective observational	Iraq	520	Age: 18 years and older (mean±SD = 56.92 ± 9.62)	190 (36.5%)	Controlled diabetes at HBA1C ˂ 7%	NA	Controlled blood sugar was among 23.4%, poor controlled blood sugar was among 76.6%.
						Uncontrolled diabetes at HBA1C ≥7%		Significant associations found between sex and hba1c level (p = 0.000), waist circumference was significantly associated with hba1c levels (p = 0.018).
						Oral anti-diabetic medications		
Almalki et al 2021 [16]	Cross-sectional	Saudi Arabia	1010	Age:26-older than 65 years (46-65 years=652(64.56%)	398 (39.41%)	Poor GC at HBA1C≥7%	Oral antidiabetic medications or insulin	Poor glycemic control presented in 49.1%.
								Risk of poor GC was associated with age with 45-65 years (P=0.0005), obesity(P=0.01), and asthma(P=0.005).
Wulandari et al 2021 [17]	Cross-sectional	Indonesia	323	Age:18-≥60 years (≥60=186(57.6%)	98 (30.3%)	Poor GC at HBA1C˃7%, good GC at HBA1C≤7%	Metformin, Glimepiride, Glibenclamide, Acarbose, Gliquidone, Insulin Glargine, Insulin Aspart, Gliclazide, Insulin Determir, Insulin Lispro.	Poor glycemic control was prevalent among 61.3%
								Age (geriatric), duration of T2DM, route of administration, number of antidiabetics, and number of other daily regular drugs significantly (P<0.05) related to glycemic control.
Espinosa et al 2021 [18]	Cross-sectional	Brazil	338	Age: 18-≥60 years (mean=58.07)	91 (26.9%)	-Poor GC at HBA1C≥7%, good GC at HBA1C˂7%, in older adults over 60 years of age, hba1c higher than 8.5% was considered inadequate control	NA	The prevalence of elevated glycated hemoglobin was 47.34%.
								Poor glycemic control was significantly associated (p<0.05) with insulin use, fasting glucose ≤70 and ≥100 mg/dl, postprandial glucose ≥180 mg/dl, no physical activity, the interaction between age group ≤59 years and the time of disease diagnosis >10 years, and presence of arterial hypertension.
Traore et al 2021 [19]	Descriptive analytical cross-sectional	Burkina Faso	270	Age:18-≥65 years (Mean=55.97)	105 (38.9%)	Poor GC at HBA1C˃7%	Mono-therapy, Bi-therapy, Insulin, oral anti-diabetic drugs alone, and dietary intake measures only	Prolonged poor control of diabetes mellitus was observed in 73.70%.
								Low level of formal education (p < 0.01), family support for diabetes mellitus management (p = 0.02), presence of abdominal obesity (p = 0.03), presence of a history of hospitalization (p <0.01), poor adherence to antidiabetic treatment (P< 0.01), and the presence of microangiopathy (p < 0.01) were the factors independently associated with prolonged poor control of T2DM.
Nigussie et al 2021 [20]	Cross-sectional	Ethiopia	394	Age:18-≥60 years (40-59 years =184(46.7%)	190 (48.2%)	Poor GC at blood sugar level˃154mg/dl	Oral anti diabetic drug, Insulin, Oral anti diabetics + insulin	The overall prevalence of poor glycemic control was 45.2%
								The risk of poor GC was increased two-fold among patients on oral anti-diabetic drugs + insulin compared to oral anti-diabetic drug alone, and patients who didn’t understand the instructions of pharmacists as well as patients who had poor practice.
Chetoui et al 2020 [21]	Cross-sectional survey	Morocco	1456	Age: 19-86 years (Mean=56.16)	388 (26.6%)	Poor GC at HBA1C ≥7%	Oral anti-diabetic alone, insulin, oral anti-diabetic + insulin, diet only	Poor glycemic control was found among 66.3%
						Good GC at HBA1C˂7%		Bivariate analysis showed that sex (p=0.010), education level (p=0.013), body mass index (p=0.048), duration of diabetes (p<0.0001) and type of therapeutic regimen (p<0.0001) were significantly associated with hba1c level.
								Multiple logistic regression analyses revealed that only a longer duration of diabetes (p=0.001) and receiving insulin therapy alone (p=0.004) or a combination of oral anti-diabetics with insulin (p<0.001) were significantly associated with inadequate glycemic control.
Achila et al 2020 [22]	Descriptive cross-sectional	Eretria	309	Age: ˂40-˃60 years (Mean=57.8 years)	163 (52.8%)	Poor GC at HBA1C≥7%	NA	Poor GC was prevalent among 76.7%.
								Poor GC was more prone among patients with abnormal waist-to-hip ratio (P=0.02), without hypertension (P=0.02), estimated glomerular filtration rate (P=0.03)
Maifitrianti et al 2020 [23]	Cross-sectional	Indonesia	126	Age:35-85 years (mean=61.46)	37 (29.4%)	-Poor GC at HBA1C at ≥7%	Single therapy, polytherapy	Poor glycemic control was prevalent among 54.8%.
						-Good GC at hba1c ˂7%		The number of antidiabetics was significantly associated with glycemic control (p<0.05). The poor glycemic control was significantly higher in patients with polytherapy (72.6%) antidiabetic compared to single antidiabetic (37.5%) (p=0.01).
Ghabban et al 2020 [24]	Retrospective	Saudi Arabia	697	Age:18-≥65 years (Mean=58.2)	444 (63.7%)	Poor GC at HBA1C=7%	Insulin, tablets, insulin+ tablets	The overall prevalence of poor glycemic control was 81.5%, whereas 18.5% of the patients showed good glycemic control.
								Higher prevalence of poor glycemic control was reported among patients with higher duration of diabetes (P=0.002), and long duration was predictive factor for poor glycemic control (P=0.003). Older patients were less prone to poor glycemic control p=0.010), the usage of combined insulin and tablet treatments was associated with a higher risk of poor glycemic control when compared to insulin only treatments (p=0.006)
Abd-Elraouf 2020 [25]	Cross-sectional	Egypt	200	Age: Mean=55.38 for poor GC group, 57 for good GC group	86 (43%)	Poor GC at HBA1C ≥7%	NA	Poor glycemic control was prevalent among 93%
						Good GC at HBA1C˂7%		There was a statistically significant association between diabetic control and diabetes duration (p < 0.001), exercise (p = 0.001), body mass index (p < 0.001). There was a statistically significant difference between those with poor and good diabetic control as regards LDL level (p < 0.001); poor GC patients tended to have high LDL (79.6%), total cholesterol level (p < 0.001), and the mean value of fasting blood sugar (p < 0.001); patients with poor glycemic control tended to have a higher level of FBS.

The included 12 articles were from nine different countries: two studies from Ethiopia [16,22], two studies from Saudi Arabia [18,26], one study from Iraq [17], two studies from Indonesia [19,25], one study from Brazil [20], one study from Burkina Faso [21], one study from Morocco [23], one study from Eretria [24], and one study from Egypt [27]. There were five studies reported from Arabian countries [17-18,23,26-27].

The total number of T2DM patients included in the studies under analysis was 5765. Among studies, the smallest sample size was 122 [16], whereas the largest sample size was 1456 [23]. The total number of males in all studies was 2257 (39.2%), whereas the total number of females was 3508 (60.8%), reflecting a higher prevalence in females. A higher number of male patients than female patients were seen in two studies [16,24]. The studies were conducted on T2DM patients 18 years old and older.

Glycemic control was determined majorly based on HbA1C as reported in 10 studies [17-21,23-27], whereas only two studies considered glycemic control based on fasting blood glucose (FBG) [16,22]. Two studies considered poor glycemic control at HbA1C ˃7% [19,21], whereas one study considered poor glycemic control at HbA1C=7% [26], and others considered it at HbA1C≥7% [17-18,20,23-25,27]. Regarding the level of FBG, one study considered good glycemic control with FBG at 70-130 mg/dL and poor glycemic control with FBG of less than 70 and more than 130 mg/dL [16]. The other study defined poor glycemic control at blood sugar levels of more than 154 mg/dL [22]. Regarding the medication regimen, three studies did not report the medication regimen of patients [20,24,27], whereas the remaining nine studies reported varied regimens.

The prevalence of poor glycemic control varied between studies based on the value of poor glycemic control considered in each study. The prevalence ranged between 45.2% and 93% [22,27]. The factors associated with poor glycemic control were varied and can be divided into four categories: personal or body-related factors, clinical factors, medication-related factors, and behavioral factors. The person or body-related factors that affected glycemic control included age [16,19-20,26], education level [16,21,23], cigarette smoking [16], gender [17,23], waist circumference [17], obesity or body mass index (BMI) [18,21,23,27], waist to hip ratio [24], family support for DM management [21], and the state of understanding the instructions of pharmacists [22].

The clinical factors that influenced the glycemic control of patients included co-morbidities, nephropathic complications of diabetes [16], asthma [18], duration of T2DM [19,23,26-27], fasting glucose [20,27], postprandial glucose, time of disease diagnosis [20], hypertension [20,24], history of hospitalization, microangiopathy [21], estimated glomerular filtration rate (eGFR) [24], and levels of cholesterol and low-density lipoprotein (LDL) [27].

Medication-related factors that affected glycemic control involved the drug regimen of Glibenclamide and metformin [16], route of administration [19], number of anti-diabetics [19,25], number of other daily regular drugs [19], insulin use [20], and the diabetes treatment regimen [22-23,26].

The behavioral factors were the least reported factors affecting the glycemic control of patients, and they included low adherence [16,21] and exercise [27].

Discussion

Poor glycemic control of DM leads to macro and microvascular complications [28]. Therefore, it is necessary to determine the factors influencing glycemic control for better glycemic control improvement. In the current analysis, we revised the previous studies and analyzed the data related to the prevalence of poor glycemic control as well as the determinant factors.

The American Diabetic Association (ADA) reports glycosylated hemoglobin (HbA1c) as the best tool for measuring glycemic control to prevent complications and reduce its cost for management [29]. In our analysis, we found that a large majority (n=10) of studies used HbA1c as an assessment for glycemic control [17-21,23-27], whereas only two studies determined glycemic control based on fasting blood glucose [16,22]. However, the 10 studies that used HbA1c for the determination of glycemic control used different cutoffs; two studies considered poor glycemic control at HbA1c more than 7% [19,21], whereas seven studies considered it poor at HbA1c≥7% [17-18,20,23-25,27] and one study considered it poor at HbA1c=7% [26]. This variation in the cutoff of poor glycemic control may lead to variation in the prevalence of poor glycemic control. Therefore, we determined the range of poor glycemic control, and we found that it was between 45.2% and 93%, reflecting high prevalence.

Earlier studies published in 2010 and 2014 from Cameron Guinea and Tanzania reported poor glycemic control among 74% and 69.7%, respectively, of diabetic patients [29-30]. A recent study from Ethiopia conducted on diabetic patients and defined good glycemic control at HbA1c less than 7% showed that more than one-half of patients (63.8%) had poor glycemic control. Poor glycemic control was significantly associated with the age of 50 years and older, female gender, being single, having high LDL, presence of diabetic peripheral neuropathy, and alcohol intake [14].

Another Ethiopian study, published in 2018, was conducted on both types 1 and 2 DM patients. It showed a higher prevalence of poor glycemic control, as 70.8% of patients showed poor glycemic control. It should be noted that the study defined poor glycemic control at mean fasting blood glucose levels above 130 mg/dL. The factors reported to be determinants of glycemic control included low education, rural residence, and longer duration of diabetes; all these factors were associated with poor glycemic control [12]. These findings reveal that the prevalence of glycemic control can vary even in the same country due to variation in the study region, the definition of the glycemic control value, and the type of diabetes. The determinant factors of glycemic control varied as well based on the evaluation of different factors between studies. However, we noted that the prevalence of poor glycemic control in the two previous studies [12,14] was high regardless of the assessment measure for glycemic control used in each study.

A recent study from Egypt was conducted on diabetic patients with no specification for the diabetes type in primary healthcare settings. It was considered that HbA1c˃7% was uncontrolled diabetes. The study showed that the education, occupation, and smoking status of patients affected diabetic control. The study further reported factors affecting glycemic control, but these factors were related to the primary healthcare physicians, and they included rural residence, participation in diabetes training, older age, longer duration since starting to deal with diabetic patients, as well as the status of following the guidelines [1].

In our analysis, all studies reported factors related to the patients, and there was no study that reported factors related to physicians affecting glycemic control. In our analysis, we categorized the determinant factors of glycemic control into four categories: personal, clinical, medication-related, and behavioral factors. The most-reported personal factors included education level, gender, body mass index, and obesity. Duration of T2DM, fasting glucose level, and hypertension were determinant factors categorized as clinical factors. The factors affecting glycemic control and assigned as medication-related factors included the number of anti-diabetics and regimen of diabetes treatment majorly. Behavioral factors were scarcely reported, and they were adherence to treatment and exercise. The factors found in our analysis were similar to the previously reported factors. However, the reported factors are dependent on the factors evaluated in each study; therefore, it is suggested to design a sheet that determines factors that should be investigated in relation to glycemic control and should be followed in further studies.

The improvement of glycemic control is necessary for T2DM patients, as there was poor glycemic control, as we found in our analysis. It was stated that the outcomes of glycemic control could be improved at the primary care level with basic interventions such as education, counseling, and continuous follow-up. Primary healthcare must involve a periodic evaluation of glycemic control and complications among T2DM patients [30].

## Conclusions

There was poor glycemic control in our study, as was reported in various studies from different regions. The factors affecting glycemic control of T2DM patients are varied. Therefore, we classified them into four categories. The major reported factors related to the patients were education level, gender, body mass index, and obesity; each can be modified except for gender. However, improving body mass index and education level can improve glycemic control. The duration of T2DM, fasting glucose level, and hypertension were determinant factors of glycemic control referred to as clinical factors. Both fasting glucose levels and hypertension can be managed by medication and good adherence to those medications, and as a result, glycemic control can be improved. The factors affecting glycemic control and assigned as medication-related factors included the number of anti-diabetics and the regimen of diabetes treatment majorly. The behavioral factors were scarcely reported, and they were adherence to treatment and exercise; these two factors can be improved by patients if they regularly practice exercise and adhere to their medication.

## References

[1] Azzam MM, Ibrahim AA, Abd El-Ghany MI (2021). Factors affecting glycemic control among Egyptian people with diabetes attending primary health care facilities in Mansoura District. Egypt J Crit Care.

[2] American Diabetes Association (2018). 2. Classification and diagnosis of diabetes: standards of medical care in diabetes-2018. Diabetes Care.

[3] International Diabetes Federation (2021). IDF Diabetes Atlas. https://idf.org/e-library/epidemiology-research/diabetes-atlas.html.

[4] American Diabetes Association (2014). Diagnosis and classification of diabetes mellitus. Diabetes Care.

[5] Hegazi R, El-Gamal M, Abdel-Hady N, Hamdy O (2015). Epidemiology of and risk factors for type 2 diabetes in Egypt. Ann Glob Health.

[6] González Clemente JM, Llauradó Cabot G (2010). Assessment of glycemic control: new insights into the evaluation of the diabetic patient. Med Clin (Barc).

[7] Monnier L, Colette C (2009). Target for glycemic control: concentrating on glucose. Diabetes Care.

[8] Lloyd A, Sawyer W, Hopkinson P (2001). Impact of long-term complications on quality of life in patients with type 2 diabetes not using insulin. Value Health.

[9] LeRoith D, Smith DO (2005). Monitoring glycemic control: the cornerstone of diabetes care. Clin Ther.

[10] Fiseha T, Alemayehu E, Kassahun W, Adamu A, Gebreweld A (2018). Factors associated with glycemic control among diabetic adult out-patients in Northeast Ethiopia. BMC Res Notes.

[11] Yigazu DM, Desse TA (2017). Glycemic control and associated factors among type 2 diabetic patients at Shanan Gibe Hospital, Southwest Ethiopia. BMC Res Notes.

[12] Abdissa D, Hirpa D (2022). Poor glycemic control and its associated factors among diabetes patients attending public hospitals in West Shewa Zone, Oromia, Ethiopia: an institutional based cross-sectional study. Metabol Open.

[13] Liberati A, Altman DG, Tetzlaff J (2009). The PRISMA statement for reporting systematic reviews and meta-analyses of studies that evaluate health care interventions: explanation and elaboration. J Clin Epidemiol.

[14] Bereda G, Bereda G (2021). The incidence and predictors of poor glycemic control among adults with type 2 diabetes mellitus in ambulatory clinic of Mettu Karl referral hospital, south western, Ethiopia: a prospective cross sectional study. Int Arch Endocrinol Clin Res.

[15] Rashad BH, Abdi BA, Naqid IA (2021). Risk factors associated with poor glycemic control in patients with type two diabetes mellitus in Zakho city. J Contemp Med Sci| Vol.

[16] Almalki ZS, Ahmed NJ, Alahmari AK (2021). Identifying the risk factors and the prevalence of poor glycemic control among diabetic outpatients in a rural region in Saudi Arabia. Int J Pharm Res.

[17] Al-Qerem W, Jarab AS, Badinjki M, Hammad A, Ling J, Alasmari F (2022). Factors associated with glycemic control among patients with type 2 diabetes: a cross-sectional study. Eur Rev Med Pharmacol Sci.

[18] Espinosa MM, Almeida VR, Nascimento VF (2021). Poor glycemic control and associated factors in diabetic people attending a reference outpatient clinic in Mato Grosso, Brazil. Invest Educ Enferm.

[19] Traoré S, Guira O, Zoungrana L (2021). Factors associated with prolonged poor glycemic control in type 2 diabetes mellitus (T2DM) patients followed in the Department of Internal Medicine at the Yalgado Ouedraogo teaching hospital, Ouagadougou (Burkina Faso). Open J Intern Med.

[20] Nigussie S, Birhan N, Amare F, Mengistu G, Adem F, Abegaz TM (2021). Rate of glycemic control and associated factors among type two diabetes mellitus patients in Ethiopia: a cross sectional study. PLoS One.

[21] Chetoui A, Kaoutar K, Elmoussaoui S, Boutahar K, El Kardoudi A, Chigr F, Najimi M (2020). Prevalence and determinants of poor glycaemic control: a cross-sectional study among Moroccan type 2 diabetes patients. Int Health.

[22] Achila OO, Ghebretinsae M, Kidane A, Simon M, Makonen S, Rezene Y (2020). Factors associated with poor glycemic and lipid levels in ambulatory diabetes mellitus type 2 patients in Asmara, Eritrea: a cross-sectional study. J Diabetes Res.

[23] Maifitrianti NW, Haro M, Lestari SF, Fitriani A (2020). Glycemic control and its factor in type 2 diabetic patients in Jakarta. Indones J Clin Pharm.

[24] Ghabban SJ, Althobaiti B, Farouk IM (2020). Diabetic complications and factors affecting glycemic control among patients with type II diabetes mellitus attending the chronic illness clinics at Tabuk, Saudi Arabia. Cureus.

[25] El-Dien Abd-Elraouf MS (2020). Factors affecting glycemic control in type II diabetic patients. Egypt J Hosp Med.

[26] Mideksa S, Ambachew S, Biadgo B, Baynes HW (2018). Glycemic control and its associated factors among diabetes mellitus patients at Ayder comprehensive specialized hospital, Mekelle-Ethiopia. Adipocyte.

[27] Khattab M, Khader YS, Al-Khawaldeh A, Ajlouni K (2010). Factors associated with poor glycemic control among patients with type 2 diabetes. J Diabetes Complications.

[28] Kamuhabwa AR, Charles E (2014). Predictors of poor glycemic control in type 2 diabetic patients attending public hospitals in Dar es Salaam. Drug Healthc Patient Saf.

[29] Ahmad NS, Islahudin F, Paraidathathu T (2014). Factors associated with good glycemic control among patients with type 2 diabetes mellitus. J Diabetes Investig.

[30] (2020). WHO package of essential noncommunicable (PEN) disease interventions for primary health care. https://www.who.int/publications/i/item/who-package-of-essential-noncommunicable-(pen)-disease-interventions-for-primary-health-care.

